# Radiomic Analysis of Magnetic Resonance Imaging for Breast Cancer with *TP53* Mutation: A Single Center Study

**DOI:** 10.3390/diagnostics15040428

**Published:** 2025-02-10

**Authors:** Jung Ho Park, Lyo Min Kwon, Hong Kyu Lee, Taeryool Koo, Yong Joon Suh, Mi Jung Kwon, Ho Young Kim

**Affiliations:** 1 Division of Breast and Endocrine Surgery, Hallym University Sacred Heart Hospital, Anyang 14068, Republic of Koreanicizm@hallym.or.kr (Y.J.S.); 2 Department of Radiology, Hallym University Sacred Heart Hospital, Anyang 14068, Republic of Korea; lyominkwon@hallym.or.kr; 3 Department of Thoracic and Cardiovascular Surgery, Hallym University Sacred Heart Hospital, Anyang 14068, Republic of Korea; 4 Department of Radiation Oncology, Hallym University Sacred Heart Hospital, Anyang 14068, Republic of Korea; 5 Department of Pathology, Hallym University Sacred Heart Hospital, Anyang 14068, Republic of Korea; 6 Department of Internal Medicine, Hallym University Sacred Heart Hospital, Anyang 14068, Republic of Korea

**Keywords:** breast neoplasms, radiomics, magnetic resonance imaging, TP53

## Abstract

**Background**: Radiomics is a non-invasive and cost-effective method for predicting the biological characteristics of tumors. In this study, we explored the association between radiomic features derived from magnetic resonance imaging (MRI) and genetic alterations in patients with breast cancer. **Methods**: We reviewed electronic medical records of patients with breast cancer patients with available targeted next-generation sequencing data available between August 2018 and May 2021. Substraction imaging of T1-weighted sequences was utilized. The tumor area on MRI was segmented semi-automatically, based on a seeded region growing algorithm. Radiomic features were extracted using the open-source software 3D slicer (version 5.6.1) with PyRadiomics extension. The association between genetic alterations and radiomic features was examined. **Results**: In total, 166 patients were included in this study. Among the 50 panel genes analyzed, only *TP53* mutations were significantly associated with radiomic features. Compared with *TP53* wild-type tumors, *TP53* mutations were associated with larger tumor size, advanced stage, negative hormonal receptor status, and HER2 positivity. Tumors with *TP53* mutations exhibited higher values for Gray Level Non-Uniformity, Dependence Non-Uniformity, and Run Length Non-Uniformity, and lower values for Sphericity, Low Gray Level Emphasis, and Small Dependence Low Gray Level emphasis compared to *TP53* wild-type tumors. Six radiomic features were selected to develop a composite radiomics score. Receiver operating characteristic curve analysis showed an area under the curve of 0.786 (95% confidence interval, 0.719–0.854; *p* < 0.001). **Conclusions**: *TP53* mutations in breast cancer can be predicted using MRI-derived radiomic analysis. Further research is needed to assess whether radiomics can help guide treatment decisions in clinical practice.

## 1. Introduction

Breast cancer is the most commonly diagnosed cancer type among women globally, with an estimated 2.3 million cases in 2022 [1], and remains the leading cause of cancer-related deaths. The incidence of breast cancer is steadily increasing, particularly among young Asian women [2]. The development of novel anti-cancer drugs has improved breast cancer treatment outcomes over the decades; however, disparities in access to care have limited the availability and use of these advanced treatments.

Breast cancer is a heterogeneous disease, classified into various subtypes based on distinct biologic characteristics. The common classification includes four intrinsic subtypes: luminal A, B, HER2-enriched, and basal-like tumors [3]. Advances in genomic analyses have enhanced subtype classification, enabling more precise treatment approaches [4]. Interestingly, each breast cancer subtype exhibits distinct radiologic phenotypes [5]. For instance, luminal A breast cancers often display an echogenic halo on imaging, reflecting peritumoral edema due to inflammation. HER2-positive breast cancers frequently present microcalcifications on mammography, while triple-negative breast cancers often appear with regular shapes and microlobulated margins on ultrasonography.

The tumor-agnostic approach, which targets genomic alterations shared across various cancer types by anti-cancer agents, has gained acceptance in the field of oncology [6,7]. Newly developed anticancer agents target common biological aberrations shared by various cancer types. For example, trastuzumab was originally developed to target HER2-positive breast cancer. Anti-HER2 agents have also been used for cancers beyond breast cancer, such as gastric cancer [8], cholangiocarcinoma [9], and salivary gland cancers [10]. Beyond HER2 amplification, other molecular targets such as BRAF mutation, RET fusion, NTRK fusion, high tumor mutation burden, or deficient mismatch repair/high microsatellite instability have been identified as biomarkers for various anti-cancer agents [6]. Tumors with known mutational status can be matched to investigational treatments or enrolled in ongoing clinical trials [11].

Targeted next-generation sequencing (NGS) is currently the most widely used method for identifying genetic alterations in clinical practice [6]. Its high efficiency and cost-effectiveness have made it a preferred alternative to conventional sequencing methods. However, the routine application of targeted NGS in clinical practice remains controversial, and its accessibility continues to be limited [12]. In this context, radiomics has emerged as a non-invasive approach for predicting the biological characteristics of tumors through imaging modalities [13]. While conventional image interpretation by radiologists can be subject to observer variability, radiomics extracts digital information from medical images and provides additional insights beyond what is observable by the human eyes. This approach has been actively applied to lung cancers and brain tumors, demonstrating its ability to predict mutation status and survival outcomes [14,15].

Artificial intelligence has significantly improved medical image processing. Deep learning algorithms enable automated medical imaging analysis, demonstrating high performance. Radiomics is anticipated to play an important role in clinical practice, in combination with advancements in artificial intelligence. Radiomics provides standardized and quantifiable features that can be interpreted by human observers, making it widely accessible and feasible, even with relatively small datasets.

Despite these advancements, few studies have explored the associations between radiomics features and genetic alterations in breast cancer. In this study, we aimed to analyze the radiomics features derived from MRI in a breast cancer cohort with known genetic profiles identified by targeted NGS.

## 2. Materials and Methods

### 2.1. Patients

We retrospectively reviewed the electronic medical records of patients with breast cancer who underwent surgery at a single tertiary hospital. Patients with available targeted NGS data between August 2018 and May 2021 were included. Clinicopathological characteristics and NGS data were collected.

### 2.2. Next Generation Sequencing Procedures

Tumor specimens were processed as previously described [16]. Specimens were obtained following surgical resection of breast tumors. Following microscopic examination, a representative tumor area was micro-dissected by an institutional pathologist. Genetic alterations analyzed included single-nucleotide variants, frameshift mutations, small insertions, deletions, and copy number alterations. The institutional gene panel targeted 50 genes including *AKT1*, *ALK*, *APC*, *ARID1A*, *ATRX*, *BRAF*, *BRCA1*, *BRCA2*, *CDH1*, *CDK4*, *CDK6*, *CDKN2A*, *CTNNB1*, *EGFR*, *HER2*, *ERBB3*, *ERBB4*, *ESR1*, *FBXW7*, *FGFR1*, *FGFR2*, *FGFR3*, *FOXA1*, *GATA3*, *H3F3A*, *IDH1*, *IDH2*, *KIT*, *KRAS*, *MAP2K1*, *MET*, *MLH1*, *MTOR*, *MYC*, *MYCN*, *NRAS*, *PDGFRA*, *PIK3CA*, *PTEN*, *RB1*, *RELA*, *RET*, *RHOA*, *RICTOR*, *ROS1*, *SMAD4*, *SMARCB1*, *SMO*, *STK11*, and *TP53*. Genomic data were visualized using the web-based Oncoprinter application (https://www.cbioportal.org/oncoprinter (accessed on 15 November 2024)).

### 2.3. Magnetic Resonance Imaging Acquisition and Tumor Segmentation

**Figure 1:**
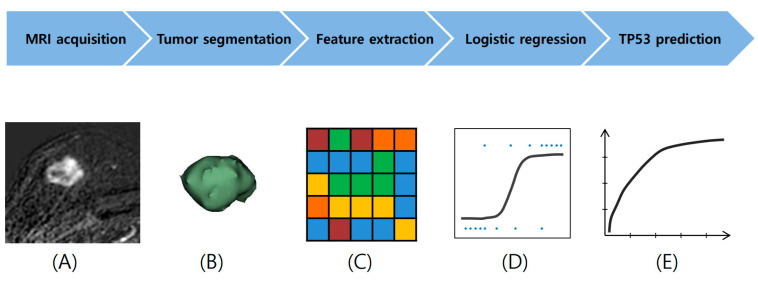
Radiomics work-flow of the study. (**A**) Substraction images were loaded. (**B**) Tumor area was segmented semi-automatically. (**C**) Radiomic features were extracted using radiomics module. (**D**) Logistic regression using radiomics features. (**E**) *TP53* mutations were predicted using ROC curve analysis.

Figure 1 illustrates the radiomics workflow. All patients underwent imaging using either of two 3T MRI scanners: Ingenia (Philips Medical Systems, Best, The Netherlands) or MAGNETOM Vida (Siemens Healthinners, Forchheim, Germany). Dynamic contrast-enhanced images were acquired before and after the intravenous injection of a gadolinium-based contrast agent. Post-contrast images were obtained at 60 s intervals following contrast administration. Subtraction images were generated by subtracting pre-contrast T1 images from the first post-contrast T1 image. Digital images were acquired as digital imaging and communication in medicine (DICOM) files from the institutional PACS server. The headers of the DICOM files were de-identified by assigning unique code numbers. The image files were loaded onto the investigator’s workstation. Tumor segmentation was performed semi-automatically, using the open-source software 3D Slicer (version 5.6.1; http://www.slicer.org (accessed on 27 December 2023)). A dedicated breast surgeon manually delineated the tumor and background areas. The tumor areas were refined using a region-growing algorithm. The tumor and background areas were manually trimmed. Only mass regions were included, and non-mass enhancement regions were excluded (Supplementary Figure S1).

### 2.4. Radiomic Feature Extraction

Radiomics features were extracted using the PyRadiomics extension in 3D slicer (version 3.0.1; Computational Imaging and Bioinformatics Lab, Harvard Medical School). A total of 107 radiomics features, comprising 14 shape-based, 18 first-order, 24 gray level co-occurrence matrix (GLCM), 14 gray level dependence matrix (GLDM), 16 gray level run length matrix (GLRLM), 16 gray level size zone matrix (GLSZM), and five neighboring gray tone difference matrix (NGTDM) features were analyzed (Supplementary Table S1).

We segmented the same tumor corresponding to that used for NGS analysis. In cases of bilateral breast cancer and multifocal or multicentric tumors, we selected only one tumor area with the most advanced stage and largest tumor, respectively.

### 2.5. Statistical Analysis

Categorical data were summarized as frequencies and percentages, while continuous data were presented as medians and ranges. The chi-squared test was used to compare categorical data. The Kolmogorov–Smirnov test was used to assess the normality of continuous data. Student’s *t*-tests were used to compare normally distributed continuous data while the Mann–Whitney U test was used to compare non-normally distributed continuous data. Statistical significance was set at *p* < 0.05.

For the comparison of the 112 radiomics features, Bonferroni correction was applied. Statistical significance was set at *p* < 5 × 10^−4^. Multivariable logistic regression with the stepwise backward elimination method was employed to select the radiomics features associated with genetic alterations. Receiver-operator characteristic (ROC) analysis was performed to evaluate the diagnostic performance of each radiomics feature. All statistical analyses were conducted using the Statistical Package for the Social Sciences Version 27.0 (IBM Corporation, Armonk, NY, USA) and R for Windows Version 4.3.1 (http://www.r-project.org (accessed on 16 June 2023)).

## 3. Results

### 3.1. Baseline Characteristics

**Figure 2:**
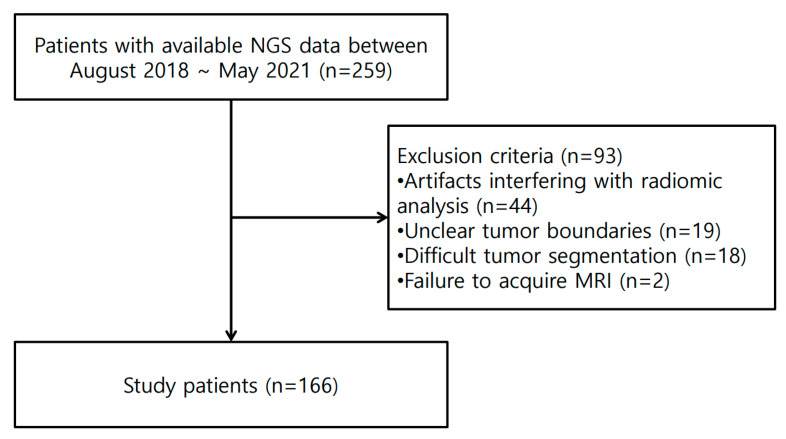
Flow-diagram of the patient selection process.

Among the 259 patients with available NGS data, we included 166 in this study (Figure 2) while excluding 93 patients for the following reasons: artifacts interfering with the radiomic analysis (*n* = 44), unclear tumor boundaries (*n* = 19), difficult tumor segmentation (*n* = 18), and failure to acquire an MRI (*n* = 2). Supplementary Table S2 provides detailed descriptions of the exclusion criteria. The excluded cases were less likely to have *TP53* mutations than those included in the study (Supplementary Table S3).

The median age of the patients was 53.5 years (range: 30–91 years). Among them, 63 (38.0%) were premenopausal, 98 (59.0%) postmenopausal, and 5 (3.0%) perimenopausal. A total of 118 (71.1%) underwent breast-conserving surgery while 48 (28.9%) underwent total mastectomy. TNM stage was distributed as follows: I (*n* = 44, 26.5%), II (*n* = 69, 41.6%), III (*n* = 46, 27.7%), and IV (*n* = 7, 4.2%). Regarding histopathological subtypes, 132 (79.5%) patients had invasive carcinomas of no special type, 10 (6.0%) had lobular carcinoma, 5 (3.0%) had micropapillary carcinoma, 4 (2.4%) had mucinous carcinoma, 4 (2.4%) had medullary carcinoma, and 11 (6.6%) had other subtypes. A total of 102 (61.4%) patients were hormone receptor-positive, and 43 (25.9%) were HER2-positive.

A total of 161 (97.0%) patients received at least one form of systemic treatment. Among the 150 (90.4%) patients who received chemotherapy, 133 (80.1%) received adjuvant chemotherapy, 10 (6.0%) received neoadjuvant chemotherapy, and 7 (4.2%) received palliative chemotherapy. Additionally, 102 (61.4%) patients received endocrine therapy and 38 (22.9%) were administered anti-HER2 therapy.

*TP53* mutations were significantly associated with larger tumor size, advanced TNM stage, high body mass index, negative hormone receptor status, and HER2 positivity (Table 1). However, no significant association was observed between age and *TP53* status.

#### BMI, Body Mass Index

**Figure 3:**
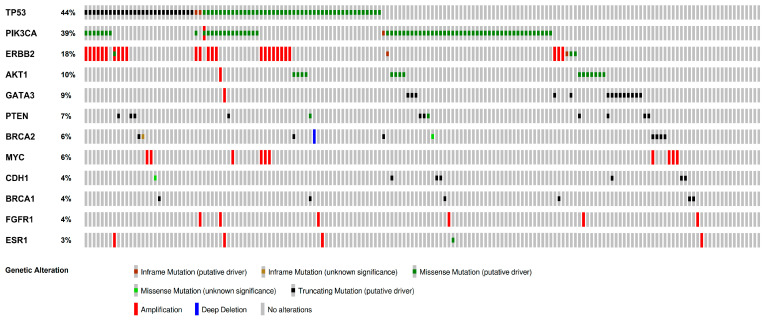
Mutational profiles of the tumors shown by Oncoprint.

Figure 3 summarizes the mutational profiles of the tumors. Among the 166 cases with available NGS data, 148 (89.2%) demonstrated significant genetic alterations, and 72 (43.4%) exhibited copy number alterations. *TP53* mutations were identified in 73 cases (44.0%), *PIK3CA* mutations in 64 cases (38.6%), *AKT1* mutations in 15 cases (9.0%), *GATA3* mutations in 14 cases (8.4%), *PTEN* mutations in 12 cases (7.2%), *BRCA2* mutations in 9 cases (5.4%), *CDH1* mutations in 8 cases (4.8%), and *BRCA1* mutations in 6 cases (3.6%). Regarding copy number alterations, *ERBB2* amplification was identified in 26 cases (15.7%), *MYC* amplification in 9 (5.4%), and *FGFR1* amplification in 6 cases (3.6%).

### 3.2. Association Between Genetic Alterations and Radiomic Features

**Figure 4:**
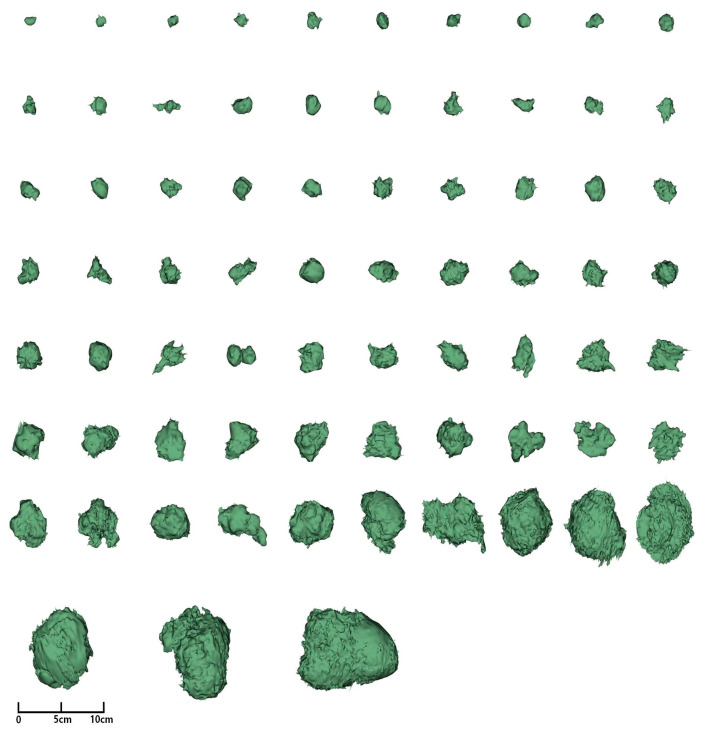
Three-dimensional views of the segmented tumors with *TP53* mutations.

Figure 4 presents the three-dimensional views of the segmented tumors with *TP53* mutations. *TP53* mutations were significantly associated with 24 radiomics features, including 14 shape-based features, 2 GLCM features, 3 GLDM features, 2 GLSZM features, 2 GLRLM features, and 1 NGTDM feature (Supplementary Table S4). Tumor size differences between *TP53* wild-type and mutant tumors were more pronounced in three-dimensional measurements (43.5 vs. 29.6, *p* = 2.35 × 10^−7^). *TP53*-mutant tumors demonstrated significantly higher Gray Level Non-Uniformity values in GLDM (2008.4 vs. 709.4, *p* = 8.49 × 10^−5^), GLRLM (1612.9 vs. 503.8, *p* = 3.21 × 10^−5^), and GLSZM (408.3 vs. 104.5, *p* = 6.61 × 10^−6^) compared with TP53 wild-type tumors. Similarly, Dependence Non-Uniformity (7524.9 vs. 2060.9, *p* = 1.91 × 10^−5^) and Run Length Non-Uniformity (32,974.8 vs. 7803.8, *p* = 5.41 × 10^−6^) were significantly elevated in *TP53*-mutant tumors. Conversely, *TP53*-mutant tumors exhibited significantly lower values for Sphericity (0.50 vs. 0.56, *p* = 2.86 × 10^−4^), Low Gray Level Emphasis (0.010 vs. 0.024, *p* = 3.31 × 10^−3^), Small Dependence Low Gray Level Emphasis (0.0015 vs. 0.0033, *p* = 1.90 × 10^−4^), and Coarseness (0.002 vs. 0.004, *p* = 2.71 × 10^−6^) compared with *TP53* wild-type tumors.

For *PIK3CA* mutations, tumors were associated with smaller Maximum 3D Diameter (33.3 vs. 37.2, *p* = 0.053), as well as lower Gray Level Non-Uniformity of GLDM (927.8 vs. 1502.1, *p* = 0.064), GLRLM (767.2 vs. 1132.2, *p* = 0.31), and GLSZM (176.0 vs. 277.0, *p* = 0.27). However, these associations were statistically not significant (Supplementary Table S5).

For *AKT1* mutations, tumors were associated with smaller Total Energy (6.12 × 10^9^ vs. 1.71 × 10^10^, *p* = 0.006), Mesh Volume (4614.1 vs. 12,152.3, *p* = 0.009), Voxel Volume (4704.4 vs. 12,307.7, *p* = 0.009), Gray Level Non-Uniformity of GLSZM (97.8 vs. 252.0, *p* = 0.011), Run Length Non-Uniformity (7297.2 vs. 20,022.9, *p* = 0.012), and Dependence Non-Uniformity (1884.6 vs. 4719.9, *p* = 0.013). However, these associations were not statistically significant (Supplementary Table S6).

*GATA3* mutations were exclusive to luminal breast cancer subtypes and these tumors were associated with higher values of Small Dependence Low Gray Level Emphasis (0.005 vs. 0.002, *p* = 9 × 10^−4^), Sphericity (0.62 vs. 0.53, *p* = 0.002), Coarseness (0.006 vs. 0.003, *p* = 0.003), Short Run Low Gray Level Emphasis (0.03 vs. 0.01, *p* = 0.004), Low Gray Level Emphasis (0.03 vs. 0.02, *p* = 0.005), and Low Gray Level Zone Emphasis (0.06 vs. 0.03, *p* = 0.006) compared with *GATA3* wild-type tumors. Conversely, tumors with *GATA3* mutations exhibited lower Maximum 3D Diameter (24.9 vs. 36.7, *p* = 0.002), Dependence Non-Uniformity (1714.3 vs. 4717.0, *p* = 0.002), Gray Level Non-Uniformity in GLDM (477.4 vs. 1354.6, *p* = 0.014), GLRLM (391.5 vs. 1046.8, *p* = 0.016), and GLSZM (88.2 vs. 252.0, *p* = 0.002). These associations demonstrated marginal statistical significance (Supplementary Table S7).

No significant associations were observed between radiomics features and other genetic alterations, including mutations in *PTEN*, *CDH1*, *BRCA1* and *BRCA2*, or amplifications of *ERBB2*, *MYC*, and *FGFR1*.

### 3.3. Prediction of Genetic Alterations by Radiomic Features

Receiver operating characteristic (ROC) curve analysis was conducted to assess the predictive power of radiomics features in identifying *TP53* mutations. Among the individual radiomics features, the Maximum 3D Diameter yielded the highest predictive accuracy, with an area under the curve (AUC) of 0.734 (Table 2). Following Maximum 3D Diameter, features such as Run Length Non-Uniformity, Gray Level Non-Uniformity of GLDM, Dependence Non-Uniformity, and Gray Level Non-Uniformity of GLRLM also exhibited acceptable predictive performances [17].

We also conducted an ROC curve analysis to evaluate the predictive power of radiomics features for identifying *PIK3CA* and *GATA3* mutations. Coarseness and Strength were associated with *PIK3CA* mutations, with neither feature demonstrating sufficient predictive ability (Table 2). For predicting GATA3 mutations, Small Dependence Low Gray Level Emphasis yielded the highest predictive power, followed by Sphericity, Coarseness, Low Gray Level Emphasis, and Low Gray Level Run Emphasis.

On multivariable logistic regression, six radiomics features—including Mean, Elongation, Skewness, Low Gray Level Emphasis, Small Dependence High Gray Level Emphasis, Gray Level Non-Uniformity of GLRLM—were selected to generate the composite radiomics score (Supplementary Table S8), which predicted *TP53* mutations with an AUC of 0.786 (95% CI, 0.719–0.854; *p* < 0.001).

## 4. Discussion

Identifying the mutational status of the tumors is essential for understanding tumor biology and guiding the use of targeted agents in precision medicine. Although targeted NGS is a common approach for determining mutational status, it is costly and requires labor-intensive interpretation. In this study, we explored the potential of radiomics as a non-invasive alternative for identifying somatic mutations in breast cancer by analyzing the association between MRI-derived features and genetic alterations identified through targeted NGS.

Among the 50 genes analyzed, only *TP53* mutations were significantly associated with MRI radiomics features. Specifically, *TP53* mutations were significantly associated with larger tumor size and higher ‘non-uniformity’ radiomics features. We demonstrated that individual or combined radiomics features could predict *TP53* mutations in breast cancer. Among the individual radiomics features, Maximum 3D Diameter was the best predictor of *TP53* mutations, posing an AUC of 0.734. When the six radiomics features were combined in a logistic regression model, the prediction of *TP53* mutations achieved an AUC of 0.786. Few studies have examined the relationship between *TP53* mutations and radiomics features in breast cancer; however, our findings align with those of previous studies. For instance, one study examining the MRI radiomics features of 229 patients with breast cancer demonstrated an AUC of 0.78 for *TP53* mutation identification using logistic regression [18]. Another study compared machine learning models using MRI radiomics features in 139 patients with breast cancer and reported an AUC of 0.74 for logistic regression [19].

*TP53* is the most commonly mutated gene in breast cancer, as reported by The Cancer Genome Atlas Network [3]. Previous studies have linked *TP53* mutations with aggressive characteristics of breast cancer, including larger tumor size, high grade, lymph node positivity, and hormone receptor negativity [20]. *TP53* mutations are considered poor prognostic markers and are associated with treatment resistance [21,22]. It has been suggested that the prognostic significance of *TP53* mutations differs according to breast cancer subtypes [23]. They were associated with poor survival in estrogen receptor-positive breast cancers and HER2-enriched subtypes but not with survival in basal-like subtypes. Routine testing for *TP53* is not performed in clinical practice because no drug directly targets *TP53* mutations [24]. However, preclinical studies have shown the potential of targeting *TP53* mutations through the reactivation of mutant p53 proteins [25].

The PI3K-Akt-mTOR pathway is the most frequently altered signaling pathway in human cancer [26]. Targeting this pathway is an active area of investigation in breast cancer. In our study, *PIK3CA* and *AKT1* mutations were associated with smaller tumor size and more uniform texture, although these associations did not meet the strict criteria for significance after Bonferroni correction.

*PIK3CA* is the second most commonly mutated gene in breast cancer [3]. *PIK3CA* mutations are established oncogenic drivers, with their clinical significance varying across breast cancer subtypes [27]. These mutations are prevalent in luminal subtypes, and are often associated with a favorable prognosis. In contrast, they account for less than 10% of triple-negative breast cancer, with the highest prevalence in the luminal androgen receptor subtype [28]. Alpelisib is the first PI3K inhibitor approved for the treatment of hormone receptor-positive, HER2-negative breast cancer [29]. *AKT1*, another key gene in the PI3K-Akt-mTOR pathway, functions as a downstream target of *PIK3CA*. Capivasertib, an AKT inhibitor, has demonstrated a significant survival benefit against hormone receptor-positive breast cancer [30].

*GATA3* mutations were associated with smaller tumor size and uniform textural features, reflecting the radiologic characteristics of luminal breast cancer. Multiple radiomics features, such as Small Dependence Low Gray Level Emphasis, Sphericity, Coarseness, Low Gray Level Emphasis, and Low Gray Level Run Emphasis had the potential to predict *GATA3* mutations. *GATA3* mutations are frequently identified in small luminal breast cancers with homogeneous enhancing patterns, indicative of a favorable prognosis. They are almost exclusively identified in luminal breast cancers, whereas *TP53* mutations are more commonly identified in hormone receptor-negative breast cancers. This finding is consistent with a previous study that reported mutual exclusivity between *GATA3* mutations and *TP53* mutations [3].

Mutations in *BRCA1* and *BRCA2* are the primary cause of hereditary breast and ovarian cancer syndrome. Patients with *BRCA* mutations can be candidates for genetic counseling, risk-reducing mastectomy, risk-reducing salpingo-oophrectomy, and treatment with poly (ADP-ribose) polymerase inhibitors [31]. On imaging, tumors with *BRCA1* mutations are more likely to appear as round and well-circumscribed lesions on imaging [32]. However, in our study, radiomic analysis alone could not differentiate *BRCA* mutational status, reinforcing that genetic sequencing remains the gold standard for identifying *BRCA* mutations. Nevertheless, a recent study suggested that radiomic analysis of contralateral breast tissue may aid in identifying *BRCA1* mutated tumors [33].

Radiomic analysis is a valuable tool for quantifying intratumoral heterogeneity, which has been linked to treatment resistance and poor prognosis [34]. In our study, *TP53* mutations were associated with higher value of ‘non-uniformity’ radiomics features, underscoring their potential as markers of intratumoral heterogeneity. Entropy measured by radiomic analysis has been proposed in previous studies as a marker of intratumoral heterogeneity [35], although this finding was not confirmed in our study. Nevertheless, our findings remain consistent with those of previous studies, demonstrating that intratumoral heterogeneity assessed via MRI can serve as a prognostic marker for breast cancer. Specifically, homogeneous enhancement patterns on MRI have been associated with a favorable prognosis of breast cancer [36]. Intratumoral heterogeneity quantified by radiomic analysis has shown predictive value for treatment response to neoadjuvant chemotherapy in breast cancer [37]. Further validation is needed to determine whether intratumoral heterogeneity measured at the radiologic level corresponds to that observed at the molecular level.

To the best of our knowledge, this is the first study to compare radiomic features of breast cancer with genetic alterations identified through targeted NGS. Our findings highlight the complementary role of radiomics in molecular diagnostics. A limitation of clinical NGS is its lack of spatial information [38]. Multi-regional sequencing and spatial transcriptomics address this limitation [39,40]; however, they are not yet available in routine clinical practice. Contrastingly, MRI provides valuable spatial information, capturing histopathological patterns such as peritumoral edema, fibrosis, and neovascularization [41]. In the era of precision medicine, the ability to non-invasively characterize tumors through imaging is increasingly relevant.

This study had several limitations. First, it was a single-center, retrospective cohort study with a limited sample size, which may have hindered the detection of possible correlations between radiomics features and genetic alterations. Additionally, our study population consisted exclusively of South Koreans, restricting the generalizability of our conclusions to other ethnic groups. Second, a significant proportion of patients were excluded because of the inapplicability of radiomic analysis, underscoring the need for consistent selection criteria in future studies utilizing radiomic analysis for breast cancer. Third, tumor segmentation was performed semi-automatically, introducing interobserver variability [42]. The use of emerging artificial intelligence-based segmentation models that provide fully automated segmentation may help address this limitation.

## 5. Conclusions

Our study demonstrated that *TP53* mutations in breast cancer can be predicted using MRI-derived radiomic analysis. This approach provides a new strategy for precision medicine in breast cancer. Further research is needed to assess whether radiomics can be utilized to guide treatment decisions in clinical practice.

## Figures and Tables

**Table 1:** Comparisons between *TP53* mutant versus wild-type tumors.

	*TP53* Wild-Type (*n* = 93)	*TP53* Mutant (*n* = 73)	*p*
Median age (range), years	54 (36–83)	53 (30–91)	0.854
Menopausal status			0.751
Premenopausal	40 (43.0%)	29 (39.7%)	
Postmenopausal	53 (57.0%)	44 (60.3%)	
BMI, kg/m^2^	23.7 (17.8–35.8)	25.3 (18.0–44.8)	0.035
Median tumor size (range), cm	2.0 (0.4–8.5)	2.5 (0.5–9.5)	0.014
TNM Stage			<0.001
I	33 (35.5%)	11 (15.1%)	
II	42 (45.2%)	27 (37.0%)	
III	15 (16.1%)	31 (42.5%)	
IV	3 (3.2%)	4 (5.5%)	
Hormone receptor			<0.001
Negative	17 (18.3%)	45 (61.6%)	
Positive	76 (81.7%)	28 (38.4%)	
HER2 status			0.001
Negative	78 (83.9%)	45 (61.6%)	
Positive	15 (16.1%)	28 (38.4%)	

**Table 2:** Predictive powers of radiomics features for identifying mutations.

Radiomic Features	AUC	95% CI	*p*
*TP53*			
Composite radiomics score	0.786	0.719–0.854	<0.001
Maximum 3D Diameter	0.734	0.659–0.810	<0.001
Run Length Non-Uniformity	0.706	0.627–0.785	<0.001
Gray Level Non-Uniformity of GLDM	0.704	0.625–0.783	<0.001
Dependence Non-Uniformity	0.694	0.614–0.773	<0.001
Gray Level Non-Uniformity of GLRLM	0.688	0.607–0.770	<0.001
*PIK3CA*			
Coarseness	0.584	0.493–0.676	0.072
Strength	0.583	0.494–0.671	0.067
*GATA3*			
Small Dependence Low Gray Level Emphasis	0.768	0.621–0.915	<0.001
Sphericity	0.742	0.604–0.881	<0.001
Coarseness	0.737	0.592–0.882	0.001
Low Gray Level Emphasis	0.725	0.580–0.870	0.002
Low Gray Level Run Emphasis	0.724	0.580–0.869	0.002

## Data Availability

The raw data supporting the conclusions of this article will be made available by the authors on request.

## Supplementary Materials

The following supporting information can be downloaded at: https://www.mdpi.com/article/10.3390/diagnostics15040428/s1, Figure S1: Detailed description of the semi-automatic segmentation process; Table S1: Radiomics features used in the study; Table S2: Detailed descriptions of the reasons for case exclusion; Table S3: Comparisons between included and excluded patients; Table S4: Radiomics features associated with *TP53* mutations; Table S5: Radiomics features associated with *PIK3CA* mutations; Table S6: Radiomics features associated with *AKT1* mutations; Table S7: Radiomics features associated with *GATA3* mutations; Table S8: Multivariable logistic regression using six radiomics features.

## Abbreviations

The following abbreviations are used in this manuscript:

AUC	Area Under the Curve
DICOM	Digital Imaging and Communication in Medicine
GLCM	Gray Level Co-occurrence Matrix
GLDM	Gray Level Dependence Matrix
GLRLM	Gray Level Run Length Matrix
GLSZM	Gray Level Size Zone Matrix
HER2	Human epidermal growth receptor 2
LASSO	Least Absolute Shrinkage and Selection Operator
MRI	Magnetic Resonance Imaging
NGS	Next Generation Sequencing
NGTDM	Neighboring Gray Tone Difference Matrix
PACS	Picture Archiving and Communication System
ROC	Receiver Operating Characteristic
