# Supplementary material for: Radiomic Analysis of Magnetic Resonance Imaging for Breast Cancer with TP53 Mutation: A Single Center Study
Source: Diagnostics (Basel). 2025 Feb 10;15(4):428. doi: 10.3390/diagnostics15040428 (PMC11854707; doi:10.3390/diagnostics15040428)
Supplement: Supplementary file 1 [file diagnostics-15-00428-s001.zip › Supplementary table.pdf]

Table S1. Radiomics features used in the study

Shape-based Features	Elongation, Flatness, Least Axis Length, Major Axis Length, Maximum 2D diameter (slice, column, row), Maximum 3D diameter, Mesh Volume, Minor Axis Length, Sphericity, Surface Area, Surface Area to Volume Ratio, Voxel Volume
First order statistics	10th Percentile, 90th Percentile, Energy, Entropy, Interquartile Range, Kurtosis, Maximum, Mean Absolute Deviation, Mean, Median, Minimum, Range, Robust Mean Absolute Deviation, Root Mean Squared, Skewness, Total Energy, Uniformity, Variance
Gray Level Co-occurrence Matrix (GLCM)	Autocorrelation, Cluster Prominence, Cluster Shade, Cluster Tendency, Contrast, Correlation, Difference Average, Difference Entropy, Difference Variance, Inverse Difference, Inverse Difference Moment, Inverse Difference Moment Normalized, Inverse Difference Normalized, Informational Measure of Correlation 1, Informational Measure of Correlation 2, Inverse Variance, Joint Average, Joint Energy, Joint Entropy, Maximal Correlation Coefficient, Maximal Probability, Sum Average, Sum Entropy, Sum of Squares
Gray Level Dependence Matrix (GLDM)	Small Dependence Emphasis, Large Dependence Emphasis, Gray Level Non-Uniformity, Dependence Non-Uniformity, Dependence Non-Uniformity Normalized, Gray Level Variance, Dependence Variance, Dependence Entropy, Low Gray Level Emphasis, High Gray Level Emphasis, Small Dependence Low Gray Level Emphasis, Small Dependence High Gray Level Emphasis, Large Dependence Low Gray Level Emphasis, Large Dependence High Gray Level Emphasis
Gray Level Run Length Matrix (GLRLM)	Gray Level Non-Uniformity, Gray Level Non-Uniformity Normalized, Gray Level Variance, High Gray Level Run Emphasis, Long Run Emphasis, Long Run High Gray Level Emphasis, Long Run Low Gray Level Emphasis, Low Gray Level Run Emphasis, Run Entropy, Run Length Non-Uniformity, Run Length Non-Uniformity Normalized, Run Percentage, Run Variance, Short Run Emphasis, Short Run High Gray Level Emphasis, Short Run Low Gray Level Emphasis
Gray Level Size Zone Matrix (GLSZM)	Small Area Emphasis, Large Area Emphasis, Gray Level Non-Uniformity, Gray Level Non-Uniformity Normalized, Size-Zone Non-Uniformity, Size-Zone Non-Uniformity Normalized, Zone Percentage, Gray Level Variance, Zone Variance, Zone Entropy, Low Gray Level Zone Emphasis, High Gray Level Zone Emphasis, Small Area Low Gray Level Emphasis, Small Area High Gray Level Emphasis, Large Area Low Gray Level Emphasis, Large Area High Gray Level Emphasis
Neighbouring Gray Tone Difference Matrix (NGTDM)	Coarseness, Contrast, Business, Complexity, Strength

Table S2. Detailed descriptions of the reasons for case exclusion

Artifacts interfering with radiomic analysis (n=44)
Extensive non-mass enhancement (38)
Large hematoma hindering segmentation (2)
Large cystic change (2)
Post-excision MRI (2)
Unclear tumor boundaries (n=19)
Lesions not identificable on T1 MRI (10)
Marked background parenchymal enhancement (4)
Faint enhancement on the tumor (3)
MRI without enhancement (1)
Occult breast cancer (1)
Difficult tumor segmentation (n=18)
Small tumor size (14)
Markedly irregular mass (4)
Failure to acquire MRI (n=2)
MRI from other hospital (1)
DICOM file error (1)

Table S3. Comparisons between included and excluded patients

	Included (n=166)	Excluded (n=93)	p
Age, *years*	55.49±11.81	54.39±11.71	0.471
BMI, *kg/m²*	24.996	24.272	0.162
Menopausal status			0.333
Premenopausal	63 (38.0%)	44 (47.3%)	
Postmenopausal	98 (59.0%)	47 (50.5%)	
Unknown	5 (3.0%)	2 (2.2%)	
Stage			0.003
I	44 (26.5%)	40 (43.0%)	
II	69 (41.6%)	20 (21.5%)	
III	46 (27.7%)	25 (26.9%)	
IV	7 (4.2%)	8 (8.6%)	
Hormone receptor			0.075
Negative	61 (36.7%)	24 (25.8%)	
Positive	105 (63.3%)	69 (74.2%)	
HER2			0.770
Negative	123 (74.1%)	67 (72.0%)	
Positive	43 (25.9%)	26 (28.0%)	
Mutation			
PIK3CA	64 (38.6%)	45 (48.4%)	0.149
TP53	73 (44.0%)	27 (29.0%)	0.023
GATA3	14 (8.4%)	9 (9.7%)	0.821
AKT1	15 (9.0%)	9 (9.7%)	1.000
PTEN	12 (7.2%)	7 (7.5%)	1.000
CDH1	8 (4.8%)	4 (4.3%)	1.000
BRCA1	6 (3.6%)	4 (4.3%)	0.749
BRCA2	9 (5.4%)	4 (4.3%)	0.776
Amplification			
ERBB2A	26 (15.7%)	10 (10.8%)	0.350
FGFR1A	6 (3.6%)	7 (7.5%)	0.234
MYCA	9 (5.4%)	2 (2.2%)	0.337

BMI, body mass index

Table S4. Radiomics features associated with *TP53* mutations

Radiomic feature	Category	*TP53* wild-type (n=93)	*TP53* mutant (n=73)	*p*
Major Axis Length	Shape-based feature	21.43276	32.66781	7.14×10^{-8}
Maximum 3D Diameter	Shape-based feature	29.57989	43.45772	2.35×10^{-7}
Maximum 2D Diameter Column	Shape-based feature	25.25584	37.11372	4.07×10^{-7}
Surface Area	Shape-based feature	2426.255	6428.87	5.26×10^{-7}
Mesh Volume	Shape-based feature	4863.597	19888.91	9.14×10^{-7}
Voxel Volume	Shape-based feature	4950.911	20117.76	9.77×10^{-7}
Maximum 2D Diameter Slice	Shape-based feature	24.91973	36.90647	1.11×10^{-6}
Maximum 2D Diameter Row	Shape-based feature	26.02364	38.17924	1.56×10^{-6}
Coarseness	NGTDM	0.004017	0.001626	2.71×10^{-6}
Minor Axis Length	Shape-based feature	17.05285	24.99688	2.99×10^{-6}
Voxel Number	Shape-based feature	10531.12	44996.34	3.08×10^{-6}
Volume Number	Shape-based feature	2.225806	3.917808	3.87×10^{-6}
Least Axis Length	Shape-based feature	13.73316	19.82248	4.49×10^{-6}
Run Length Non-Uniformity	GLRLM	7803.793	32974.83	5.41×10^{-6}
Gray Level Non-Uniformity	GLSZM	104.5397	408.2527	6.61×10^{-6}
Dependence Non-Uniformity	GLDM	2060.926	7524.869	1.91×10^{-5}
Gray Level Non-Uniformity	GLRLM	503.7892	1612.873	3.21×10^{-5}
Gray Level Non-Uniformity	GLDM	709.3825	2008.436	8.49×10^{-5}
Size Zone Non-Uniformity	GLSZM	1480.752	5432.235	1.83×10^{-4}
Small Dependence Low Gray Level Emphasis	GLDM	0.003336	0.001578	1.90×10^{-4}
Inverse Difference Moment Normalized	GLCM	0.980754	0.986293	2.08×10^{-4}
Surface Volume Ratio	Shape-based feature	0.681955	0.549997	2.25×10^{-4}
Sphericity	Shape-based feature	0.563408	0.501815	2.86×10^{-4}
Inverse Difference Normalized	GLCM	0.911081	0.924597	3.09×10^{-4}
Large Dependence High Gray Level Emphasis	GLDM	6322.878	9394.22	5.77×10^{-4}
Total Energy	First-order statistics	7.62×10^{9}	2.7×10^{10}	1.06×10^{-3}
Dependence Entropy	GLDM	6.927402	7.241509	1.26×10^{-3}
Energy	First-order statistics	2.16×10^{10}	7.12×10^{10}	1.64×10^{-3}
Small Area Low Gray Level Emphasis	GLSZM	0.018933	0.008868	2.25×10^{-3}
Short Run Low Gray Level Emphasis	GLRLM	0.021419	0.008885	2.48×10^{-3}
Low Gray Level Zone Emphasis	GLSZM	0.038356	0.016166	2.62×10^{-3}
Low Gray Level Run Emphasis	GLRLM	0.024875	0.010159	3.07×10^{-3}
Low Gray Level Emphasis	GLDM	0.024229	0.009913	3.31×10^{-3}
Zone Entropy	GLSZM	6.396189	6.806975	3.95×10^{-3}

Range	First-order statistics	1042.269	1387.315	4.49×10^{-3}
Long Run Low Gray Level Emphasis	GLRLM	0.050191	0.018587	7.00×10^{-3}
Long Run High Gray Level Emphasis	GLRLM	843.1357	1198.832	8.25×10^{-3}
Large Area High Gray Level Emphasis	GLSZM	423726.6	1300177	1.08×10^{-2}
Correlation	GLCM	0.603178	0.644846	1.38×10^{-2}
High Gray Level Run Emphasis	GLRLM	699.203	989.0529	1.61×10^{-2}
High Gray Level Emphasis	GLDM	701.0378	989.9902	1.72×10^{-2}
Large Dependence Low Gray Level Emphasis	GLDM	1.203588	0.428586	1.85×10^{-2}
High Gray Level Zone Emphasis	GLSZM	678.6642	971.3954	2.04×10^{-2}
Small Area High Gray Level Emphasis	GLSZM	457.5844	663.7655	2.13×10^{-2}
Complexity	GLCM	4212.371	6545.984	2.24×10^{-2}
Autocorrelation	NGTDM	729.2092	1017.515	2.24×10^{-2}
Joint Average	GLCM	21.49503	26.48017	2.26×10^{-2}
Sum Average	GLCM	42.99006	52.96034	2.26×10^{-2}
Maximal Correlation Coefficient	GLCM	0.639256	0.677103	2.90×10^{-2}
Run Entropy	GLRLM	4.84944	5.124103	4.07×10^{-2}

GLCM, Gray Level Co-occurrence Matrix; GLSZM, Gray Level Size Zone Matrix; GLRLM, Gray Level Run Length Matrix; NGTDM, Neighbouring Gray Tone Difference Matrix; GLDM, Gray Level Dependence Matrix

Table S5. Radiomics features associated with *PIK3CA* mutations

Radiomic feature	Category	*PIK3CA* wild-type (n=102)	*PIK3CA* mutant (n=64)	*p*
Minor Axis Length	shape-based feature	21.65916	18.7727	0.021213
Mesh Volume	shape-based feature	12738.97	9450.474	0.02748
Voxel Volume	shape-based feature	12896.58	9587.194	0.028425
Surface Area	shape-based feature	4596.991	3532.127	0.034425
Least Axis Length	shape-based feature	17.3516	14.9119	0.035282
Major Axis Length	shape-based feature	27.4666	24.63131	0.036157
Maximum 2D Diameter Slice	shape-based feature	31.72497	27.74624	0.038582
Surface Volume Ratio	shape-based feature	0.596984	0.666864	0.048298
Maximum 3D Diameter	shape-based feature	37.16034	33.32794	0.053288
Gray Level Non-Uniformity	GLDM	1502.072	927.7667	0.063888
Maximum 2D Diameter Row	shape-based feature	32.95107	28.84803	0.113653
Coarseness	NGTDM	0.002503	0.003703	0.119697
Maximum 2D Diameter Column	shape-based feature	31.94533	28.11986	0.127456
Total Energy	first order statistics	1.93×10^{10}	1.1×10^{10}	0.167443
Volume Num	shape-based feature	3.303922	2.4375	0.202814
Run Variance	GLRLM	0.225488	0.177124	0.205021
Dependence Variance	GLDM	6.132576	5.038058	0.211127
Energy	first order statistics	5.13×10^{10}	3.07×10^{10}	0.217671
Busyness	NGTDM	4.492386	2.403799	0.228737
Long Run Emphasis	GLRLM	1.573439	1.474987	0.261703
Gray Level Non-Uniformity	GLSZM	277.045	176.0319	0.271144
Strength	NGTDM	1.477206	1.91234	0.285889
Size Zone Non-Uniformity	GLSZM	3748.168	2374.218	0.293058
Cluster Shade	GLCM	-1131.9	-622.619	0.299551
Gray Level Non-Uniformity	GLRLM	1132.242	767.241	0.308011
Large Dependence Emphasis	GLDM	28.07637	23.62027	0.31156
Sphericity	shape-based feature	0.529627	0.546992	0.314844
Dependence Non-Uniformity	GLDM	5130.875	3400.504	0.316696
Voxel Num	shape-based feature	29737.34	19233.09	0.322606
Large Area Low Gray Level Emphasis	GLSZM	1949.176	154.7416	0.323496
Run Length Non-Uniformity	GLRLM	21839.81	14144.6	0.323883
LargeAreaEmphasis	GLSZM	24641.67	6172.617	0.326191

Table S6. Radiomics features associated with *AKT1* mutations

Radiomic feature	Category	*AKT1* wild-type (n=151)	*AKT1* mutant (n=15)	*p*
Total Energy	first order statistics	1.71×10^{10}	6.12×10^{9}	0.006904272
Mesh Volume	shape-based feature	12152.28	4614.106	0.009165653
Voxel Volume	shape-based feature	12307.72	4704.379	0.009309702
Voxel Num	Mask original	27275.07	9706.133	0.010500789
Gray Level Non-Uniformity	GLSZM	252.0383	97.7901	0.010985138
Run Length Non-Uniformity	GLRLM	20022.9	7297.195	0.012420255
Dependence Non-Uniformity	GLDM	4719.946	1884.641	0.012934602
Energy	first order statistics	4.59×10^{10}	1.77×10^{10}	0.012980413
Gray Level Non-Uniformity	GLRLM	1046.128	441.7838	0.01347558
Gray Level Non-Uniformity	GLDM	1355.356	528.6465	0.0146027
Volume Number	Mask original	3.092715	1.733333	0.018221844
Size Zone Non-Uniformity	GLSZM	3403.444	1356.204	0.020239502
Surface Area	shape-based feature	4347.981	2560.269	0.02544791
Coarseness	NGTDM	0.003074	0.001873	0.041893268
Least Axis Length	shape-based feature	16.64801	14.02501	0.048565988
MCC	GLCM	0.653012	0.684964	0.050571642
Small Dependence Low Gray Level Emphasis	GLDM	0.002636	0.001822	0.122883289
Minor Axis Length	shape-based feature	20.79798	18.01283	0.139368243
Maximum 2D Diameter Slice	shape-based feature	30.55525	26.52431	0.162528713
Large Area High Gray Level Emphasis	GLSZM	855614.8	341442.4	0.182634776
Minimum	Image original	-86.0132	-53.2	0.184257764
Maximum 2D Diameter Column	shape-based feature	30.79216	27.23187	0.210311283
Correlation	GLCM	0.619138	0.645293	0.211881648
Imc2	GLCM	0.772023	0.795705	0.222324363
Elongation	shape-based feature	0.794198	0.75142	0.238582482
Dependence Entropy	GLDM	7.050946	7.212383	0.261256994
Zone Variance	GLSZM	18545.5	4981.959	0.290163586
Large Area Emphasis	GLSZM	18738.32	5267.476	0.295980635
Flatness	shape-based feature	0.643022	0.601519	0.298512435
Large Area Low Gray Level Emphasis	GLSZM	1363.328	190.4561	0.339971709
Maximum 3D Diameter	shape-based feature	35.96601	32.83167	0.373908239
Strength	NGTDM	1.671389	1.378997	0.433243204
Major Axis Length	shape-based feature	26.55305	24.56574	0.459541258

Table S7. Radiomic features associated with *GATA3* mutations

Radiomic feature	Category	*GATA3* wild-type (n=152)	*GATA3* mutant (n=14)	*p*
Small Dependence Low Gray Level Emphasis	GLDM	0.00229752	0.005443326	0.000935
Maximum 3D Diameter	shape-based feature	36.6759004	24.90043649	0.002175
Dependence Non-Uniformity	GLDM	4716.98039	1714.319545	0.002217
Run Length Non-Uniformity	GLRLM	19971.6356	6944.757088	0.002442
Gray Level Non-Uniformity	GLSZM	251.902898	88.24240795	0.00249
Sphericity	shape-based feature	0.52842053	0.622106313	0.00274
Coarseness	NGTDM	0.00265474	0.006339824	0.003436
Idmn	GLCM	0.98394837	0.974955849	0.003701
Voxel Num	Mask original	27212.3684	9131.928571	0.003841
Surface Area	shape-based feature	4389.81077	1978.42818	0.004059
Short Run Low Gray Level Emphasis	GLRLM	0.01447882	0.031414445	0.004449
Major Axis Length	shape-based feature	27.0724699	18.78441698	0.005051
Low Gray Level Emphasis	GLDM	0.01643849	0.03416206	0.005427
Maximum 2D Diameter Row	shape-based feature	32.2020789	22.32624338	0.005525
Low Gray Level Run Emphasis	GLRLM	0.01682875	0.035501195	0.005624
Volume Num	Mask original	3.125	1.285714286	0.005779
Idn	GLCM	0.91856513	0.900303015	0.005933
Maximum 2D Diameter Slice	shape-based feature	30.9776394	21.65041922	0.006201
Low Gray Level Zone Emphasis	GLSZM	0.02611862	0.055515577	0.006951
Size Zone Non-Uniformity	GLSZM	3407.15036	1169.730354	0.007198
Small Area Low Gray Level Emphasis	GLSZM	0.01330824	0.027519902	0.007582
Maximum 2D Diameter Column	shape-based feature	31.2066913	22.47696508	0.009155
Minor Axis Length	shape-based feature	21.0417033	15.16773901	0.010131
Voxel Volume	shape-based feature	12322.0522	4005.663688	0.010131
Mesh Volume	shape-based feature	12164.9435	3938.122859	0.010302
Least Axis Length	shape-based feature	16.7977652	12.21173463	0.012567
Long Run Low Gray Level Emphasis	GLRLM	0.03407848	0.060336363	0.012985
Gray Level Non-Uniformity	GLDM	1354.63969	477.3710044	0.014271
Gray Level Non-Uniformity	GLRLM	1046.78248	391.5111123	0.015979
Large Dependence High Gray Level Emphasis	GLDM	7928.05466	4910.103611	0.017061
Energy	first order statistics	4.5174×10^{10}	24055553425	0.020896
Total Energy	first order statistics	1.6866×10^{10}	8288106261	0.023974
Long Run High Gray Level Emphasis	GLRLM	1036.3077	600.5421144	0.029109

Table S8. Multivariable logistic regression using six radiomics features

	B	Exp(B)	95% CI	*p*
Mean	-0.002	0.998	0.998–0.999	<0.001
Elongation	-4.585	0.010	0.000–0.235	0.004
Skewness	-1.432	0.239	0.077–0.744	0.014
Low Gray Level Emphasis	-58.451	0.000	0.000–0.000	0.000
Small Dependence High Gray Level Emphasis	0.001	1.001	1.000–1.003	0.032
Gray Level Non-Uniformity of GLRLM	0.001	1.001	1.000–1.001	0.001
Constant	4.783	119.468		0.001
