# Supplementary figures and images for: Radiomic Analysis of Magnetic Resonance Imaging for Breast Cancer with TP53 Mutation: A Single Center Study
Source: Diagnostics (Basel). 2025 Feb 10;15(4):428. doi: 10.3390/diagnostics15040428 (PMC11854707; doi:10.3390/diagnostics15040428)

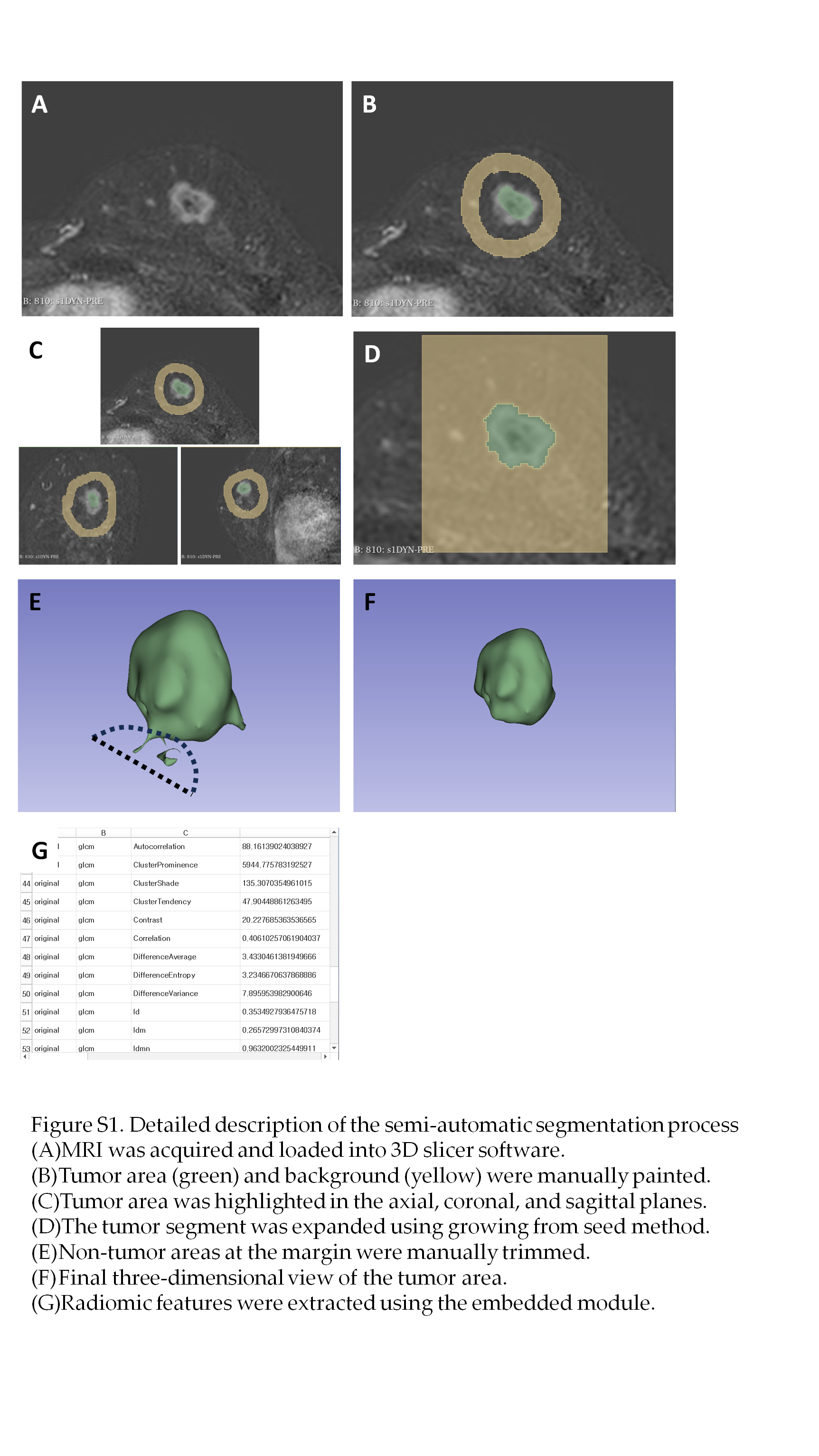

Supplement: Supplementary file 1 [file diagnostics-15-00428-s001.zip › Supplementary Figure S1.png]
